# Stat3 and CCAAT enhancer–binding protein β (C/ebpβ) activate Fanconi C gene transcription during emergency granulopoiesis

**DOI:** 10.1074/jbc.RA117.000528

**Published:** 2018-01-30

**Authors:** Chirag A. Shah, Larisa Broglie, Liping Hu, Ling Bei, Weiqi Huang, Danielle B. Dressler, Elizabeth A. Eklund

**Affiliations:** From the ‡ Feinberg School of Medicine, Northwestern University, Chicago, Illinois 60605,; the § Jesse Brown Veterans Affairs Medical Center, Chicago, Illinois 60612, and; the ¶ Children's Hospital of Wisconsin, Medical College of Wisconsin, Milwaukee, Wisconsin 53213

**Keywords:** innate immunity, STAT transcription factor, DNA repair, CCAAT-enhancer-binding protein (C/EBP), stress response, Stat3

## Abstract

Interferon consensus sequence–binding protein (Icsbp) is required for terminating emergency granulopoiesis, an episodic event responsible for granulocyte production in response to infections and a key component of the innate immune response. Icsbp inhibits the expression of Stat3 and C/ebpβ, transcription factors essential for initiating and sustaining granulopoiesis, and activates transcription of Fanconi C (*FANCC*), a DNA repair protein. In prior studies, we noted accelerated bone marrow failure in Fancc^−/−^ mice undergoing multiple episodes of emergency granulopoiesis, associated with apoptosis of bone marrow cells with unrepaired DNA damage. Additionally, we found increased expression of Fanconi C and F proteins during emergency granulopoiesis. These findings suggest that Icsbp protects the bone marrow from DNA damage by increasing activity of the Fanconi DNA repair pathway, but the mechanisms for *FANCC* activation during initiation of emergency granulopoiesis are unclear. In this study, we observed that Stat3 and C/ebpβ activate *FANCC* transcription and contribute to DNA repair. Our findings indicate that FancC expression is increased during Stat3- and C/ebpβ-induced initiation of emergency granulopoiesis by these transcription factors and is maintained through termination by Icsbp. Our work reveals that Stat3- and C/ebpβ-mediated FancC expression is a critical component for initiating and sustaining key innate immune responses.

## Introduction

The Fanconi DNA repair pathway rescues collapsed or stalled replication forks during S phase of the cell cycle, protects chromatin common fragile sites during DNA replication, and effects repair DNA interstrand cross-links (1–6). Fanconi proteins are categorized in three groups: core components, substrates, and effectors. Assembly of the core components (Fanconi A, B, C, E, F, L, M) into a complex with ubiquitin ligase activity is the first step of activating the Fanconi pathway. This complex activates the substrate components (Fanconi D2 and I) through monoubiquitination. These substrate proteins recruit effectors to sites of DNA damage (Fanconi D1, J, O, P, and Rad51) (1, 3). Fanconi anemia (FA)^3^ is an inherited disorder because of mutation of a gene in the Fanconi repair pathway. FA is clinically variable but classically involves skeletal abnormalities, bone marrow failure (BMF) during childhood, and progression to acute myeloid leukemia in subjects surviving BMF (1, 7, 8). In prior studies, we found that BMF and/or clonal progression were accelerated in a murine model of FA (*Fancc*^−/−^ mice) by repeated stimulation of an emergency (stress) granulopoiesis response (9).

Emergency granulopoiesis is an episodic process for the production of granulocytes in response to infectious challenge and a key component of the innate immune response (10, 11). In contrast, steady-state granulopoiesis is a continuous process for replacement of granulocytes lost to normal programmed cell death. Studies using murine models with gene disruptions demonstrated that emergency granulopoiesis requires IL1β and IL1β-induced expression of G-CSF at levels that are 10-fold greater than the steady state (11, 12). In additional murine gene disruption studies, Stat3 and C/ebpβ were found to be necessary for initiation and maintenance of the emergency granulopoiesis response (13, 14). Also, such studies determined that termination of emergency granulopoiesis requires the leukemia suppressor Icsbp (also known as interferon regulatory factor 8 (Irf8)) and the proto-oncogene HoxA10 (15, 16). In contrast, steady-state granulopoiesis requires Stat5 and C/ebpα and is facilitated by G-CSF and GM-CSF (12).

In prior studies, we found that Icsbp activated *FANCC* and *FANCF* gene transcription during emergency granulopoiesis (encoding Fanconi C and F, respectively (FancC and FancF)) (9, 17). This was associated with Icsbp-dependent protection of bone marrow cells from DNA damage during *in vivo* stimulation of emergency granulopoiesis in mice or *ex vivo* stimulation of bone marrow cells with IL1β or G-CSF (9, 17). Also, we determined that Icsbp is required for decreased expression of Stat3 and C/ebpβ during termination of the emergency granulopoiesis response (15).

Emergency granulopoiesis is studied in mice by intraperitoneal (i.p.) injection with pathogens (*Candida albicans* or encapsulated bacteria) or an antigen/adjuvant mixture referred to as “Alum” (ovalbumin antigen and aluminum hydroxide adjuvant) (9, 11, 15, 18). In WT mice, this results in immediate release of mature granulocytes from the bone marrow, followed by enhanced commitment of hematopoietic stem cells to granulocytes, with maximal expansion of granulocyte/monocyte progenitors and differentiating granulocytes in the bone marrow by 2 weeks (9, 15). By 4 weeks, the process has terminated and steady state resumes.

We found that injecting Alum every 4 weeks repeated this process in WT mice without resulting in morbidity or mortality (9, 15). We performed repeated induction of emergency granulopoiesis to mimic repeated infectious challenges because of environmental exposure to bacterial and fungal pathogens, which occur on an ongoing basis in humans.

Although Alum injection induced immediate granulocyte release from the bone marrow of *Fancc*^−/−^ mice, there was no subsequent expansion of myeloid progenitors or production of mature granulocytes (9). Instead, repeated emergency granulopoiesis attempts resulted in progressive pancytopenia and death in most *Fancc*^−/−^ mice, associated with apoptosis of bone marrow hematopoietic stem cells and progenitors (9). Surviving mice developed clonal progression with a rapid rise of myeloid blasts in the bone marrow (9).

These studies suggest that Icsbp protects the bone marrow from DNA-damage as emergency granulopoiesis is terminating by increasing the activity of the Fanconi DNA repair pathway (9, 15, 17). However, the mechanisms to increase *FANCC* transcription during initiation of emergency granulopoiesis or at peak granulocyte production are not clear.

In this work, we investigate the roles of Stat3 and C/ebpβ in activation of the *FANCC* gene promoter. We determine that Stat3 and C/ebpβ increase *FANCC* transcription during initiation of emergency granulopoiesis, that Icsbp cooperates with these two transcription factors for maximal *FANCC* transcription during peak granulocyte production, and that Icsbp maintains *FANCC* expression as Stat3 and C/ebpβ levels fall during termination of this process. We also implicate a sustained increase in Stat3 and C/ebpβ proteins as the major mechanism for this activity during initiation and peak emergency granulopoiesis.

## Results

### Stat3 and C/ebpβ activate separate FANCC promoter cis elements

In prior studies, we identified an Icsbp binding cis element in the proximal *FANCC* promoter (−48 to −56 bp) (9). We hypothesize that Icsbp protects bone marrow stem and progenitor cells from DNA damage during termination of emergency granulopoiesis. Because Stat3 and C/ebpβ are required to initiate and sustain emergency granulopoiesis, we considered the possibility that these transcription factors activate the *FANCC* promoter early in this process.

**Figure 1.**
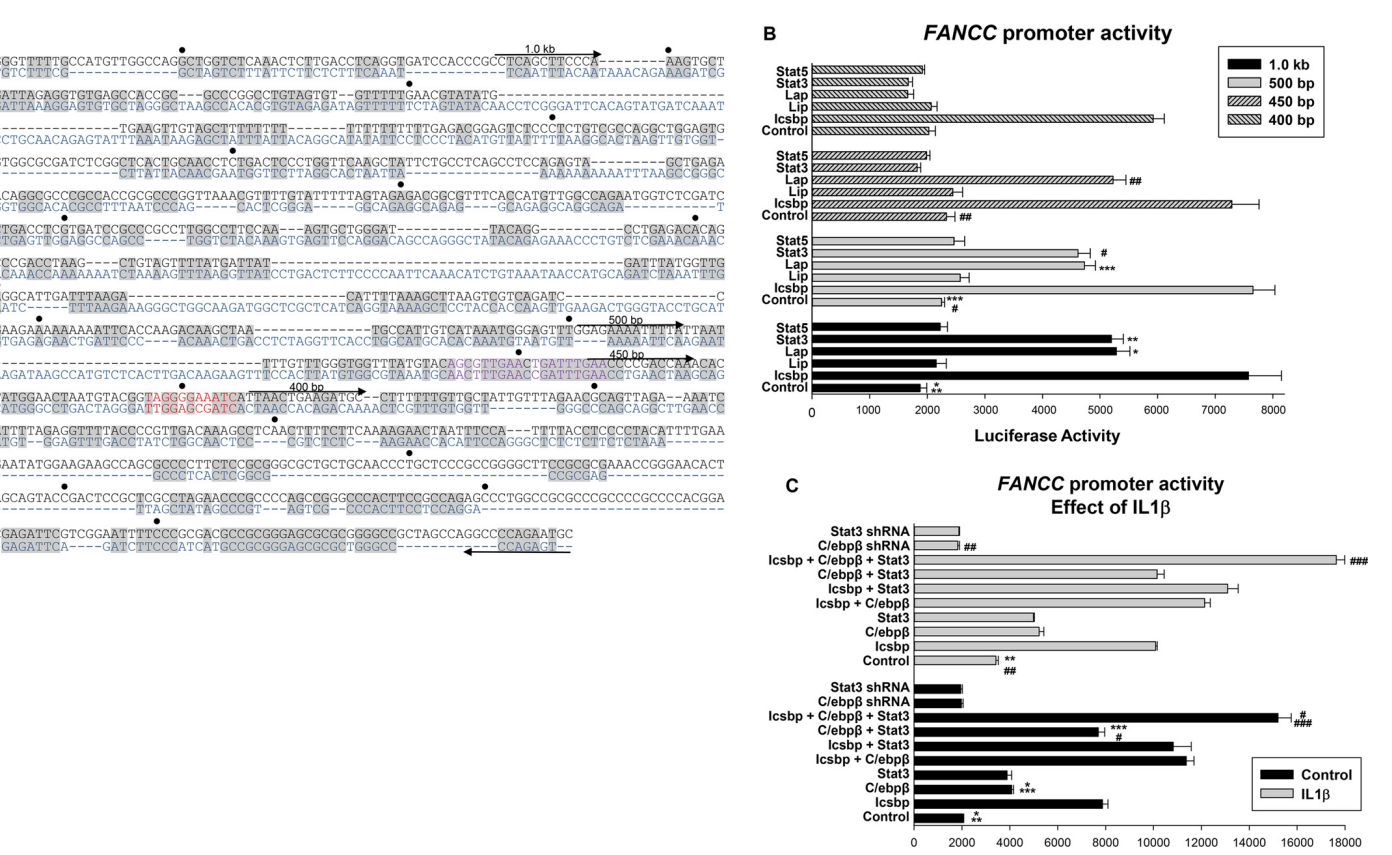
**C/ebpβ and Stat3 activate the *FANCC* promoter in myeloid cells.** *A*, alignment of the human and murine *FANCC* promoters identified conserved consensus sequences for Stat and C/ebp binding. The human sequence is shown in *black* and the murine in *blue*. Conserved regions are indicated in *gray*. The tandem Stat consensus is shown in *purple* and the C/ebp consensus in *red*. Truncations for reporter assays are indicated. *B*, Stat3 and C/ebpβ-Lap activate discrete regions of the *FANCC* promoter, but C/ebpβ-Lip and Stat5 do not influence *FANCC* promoter activity. U937 cells transfected with a 1-kb *FANCC* promoter construct or truncated derivatives thereof were co-transfected with DNA plasmids driving the expression of each of the three different transcription factors indicated. Reporter constructs with truncations of the *FANCC* promoter were assayed for the effect of overexpressed Stat3, Stat5, C/ebpβ (Lap or Lip isoform), Icsbp (positive control), or empty vector. Statistically significant differences are indicated by *, **, ***, #, or ## (*p* < 0.01, *n* = 6 for all comparisons). *C*, Stat3, C/ebpβ-Lap, and Icsbp are not redundant for *FANCC* promoter activation. The 1.0 kb of *FANCC* promoter/luciferase reporter construct was assayed in U937 transfectants for the effect of combinations of overexpressed Stat3, C/ebpβ (Lap), or Icsbp or shRNA knockdown of Stat3 or C/ebpβ. Some transfectants were differentiated with IL1β prior to analysis. Statistically significant differences are indicated by *, **, ***, #, or ## (*p* < 0.01, *n* = 6 for all comparisons).

To investigate this hypothesis, we assayed the promoter activity of a set of reporter constructs with −1.0 kb to −400 bp of the *FANCC* 5′ flank linked to a firefly luciferase reporter (or an empty control reporter vector) (Fig. 1*A*). We determined the activity of these constructs in U937 myeloid cells co-transfected with vectors to express Stat3 or C/ebpβ (*versus* an empty control expression vector). For these studies, we considered two isoforms of C/ebpβ: Lap and Lip. The former is a transcriptional activator, and the shorter Lip isoform is an antagonist of Lap (19). Because Stat3 and Stat5 share similar binding site consensus sequences, we tested both proteins. Icsbp was a positive control for activation of the *FANCC* promoter. All cells were co-transfected with an internal control reporter vector (with a CMV promoter and *Renilla* luciferase reporter) to normalize for transfection efficiency.

We found that Stat3, C/ebpβ-Lap, and Icsbp each significantly increased the reporter activity from constructs with 1.0 kb or 500 bp of the *FANCC* promoter (*p* < 0.001, *n* = 6) (Fig. 1*B*). Both Icsbp and C/ebpβ-Lap activated a construct with 450 bp of the *FANCC* promoter (*p* < 0.001, *n* = 6) but Stat3 did not. This suggested the presence of a Stat3-influenced cis element between 450 and 500 bp in the *FANCC* promoter.

Further truncation of the promoter to 400 bp abolished activation by C/ebpβ-Lap, identifying a potential cis element between −450 and −400 bp (Fig. 1*B*). None of the *FANCC* promoter constructs were activated or repressed by Stat5 or C/ebpβ-Lip. None of these proteins influenced the activity of an empty reporter control vector, and this was subtracted as background.

Stat3 and C/ebpβ are required for emergency granulopoiesis, and IL1β is the essential cytokine for this process. Therefore, we tested the effect of Stat3 and C/ebpβ on *FANCC* promoter activity during differentiation with IL1β (11). We assayed the activity of the 1.0 kb *FANCC* promoter/luciferase reporter construct in U937 cells co-transfected with vectors to overexpress or knock down Stat3 or C/ebpβ-Lap. We found that IL1β significantly increased *FANCC* promoter activity with or without overexpression of Stat3 or C/ebpβ-Lap (*p* < 0.0001, *n* = 6) (Fig. 1*C*). We also found that knockdown of either Stat3 or C/ebpβ significantly decreased *FANCC* promoter activity (*p* < 0.0001, *n* = 6), but only in IL1β-treated transfectants. This was consistent with a role for endogenous Stat3 and C/ebpβ in *FANCC* promoter activation during IL1β-induced differentiation.

We investigated possible additive or cooperative effects of these transcription factors on the *FANCC* promoter despite interacting with discrete binding sites. For these studies, we co-transfected U937 cells with the 1.0 *FANCC* promoter/firefly luciferase reporter vector and various combinations of vectors to overexpress Stat3, C/ebpβ-Lap, or Icsbp. Cells were co-transfected with the internal control reporter vector described above. We found that combination of any two of these transcription factors was additive for *FANCC* promoter activity (*p* < 0.001, *n* = 6), and the effect of all three was greater than any two (*p* < 0.001, *n* = 6) (Fig. 1*C*). The total amount of expression vector was maintained at a constant level in these studies (*e.g.* there is half the amount of Stat3 plasmid in Stat3 + C/ebpβ experiments compared with experiments with Stat3 alone). Therefore, these studies indicated cooperation, rather than redundancy, between the three transcription factors.

### Stat3 and C/ebpβ bind to and activate discrete FANCC promoter cis elements

To confirm that Stat3 and C/ebpβ activated cis elements within the promoter regions defined above, we performed a sequence analysis (using Vista Tools for Comparative Software) (20, 21). We identified a tandem Stat consensus sequence from −442 to −464 bp in the *FANCC* promoter (separated by 2 bp). These sites were conserved in mouse and human but slightly divergent from the derived consensus for Stat3 binding (consensus, 5′-T(T/G)N_4–5_GAA-3′; proximal *FANCC* site, 5′-TGATTTGAA-3′; distal site, 5′-AGCGTTGAA-3′) (23, 24). We generated a reporter construct with one copy of this tandem site linked to a minimal promoter (−438 to −474 bp from the *FANCC* promoter in the pGL3-promoter vector). Additional constructs were generated with mutation of the proximal, distal, or both putative Stat3 binding sites. Reporter activity was assayed in U937 cells co-transfected with vectors to express Stat3, Stat5, or an empty control vector (and a reporter vector to function as an internal control as described above).

**Figure 2.**
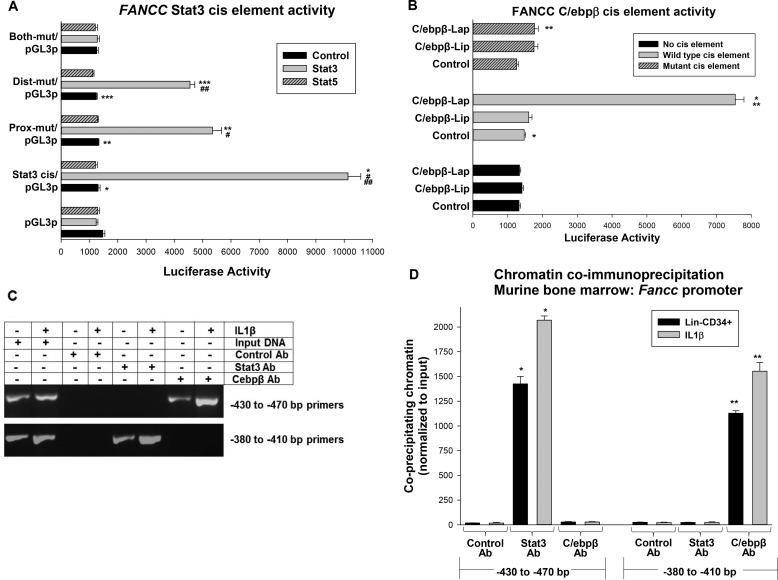
**Stat3 and C/ebpβ-Lap interact with discrete *FANCC* promoter cis elements.** *A*, Stat3 activates tandem cis elements in the *FANCC* promoter. U937 cells were transfected with a minimal promoter/luciferase reporter construct (designated pGL3p) with −438 to −474 bp of *FANCC* 5′ flank or constructs with mutation in one or both of the Stat binding consensus sequences (or the minimal promoter/luciferase reporter control vector). Cells were co-transfected with vectors to overexpress Stat3 or Stat5 (*versus* control vector). Statistically significant differences are indicated by *, **, ***, #, or ## (*p* < 0.01, *n* = 6 for all comparisons). *B*, C/ebpβ-Lap activates a cis element in the *FANCC* promoter but C/ebpβ-Lip does not. U937 cells were transfected with a minimal promoter/luciferase reporter construct with three copies of the −385 to −408 bp of *FANCC* 5′ flank or a construct with mutation of the C/ebp consensus sequence (or the minimal promoter/luciferase reporter control vector). Cells were co-transfected with vectors to overexpress the Lap or Lip forms of C/ebpβ (*versus* the control vector). Statistically significant differences are indicated by * or ** (*p* < 0.001, *n* = 6 for all comparisons). *C*, Stat3 and C/ebpβ bind to the *FANCC* promoter. Murine bone marrow myeloid progenitor cells were analyzed by chromatin immunoprecipitation with antibodies to Stat3 or C/ebpβ or irrelevant control antibody. Some cells were differentiated for 24 h with IL1β prior to analysis. Co-precipitating chromatin was amplified by PCR with primers flanking the cis elements activated by Stat3 or C/ebpβ and separated by acrylamide gel electrophoresis. *D*, IL1β increases Stat3 or C/ebpβ binding to *FANCC* promoter cis elements. Some co-immunoprecipitated chromatin was analyzed by quantitative real-time PCR (data are presented using the standard curve method). Statistically significant differences are indicated by * or ** (*p* < 0.01, *n* = 4 for all comparisons).

We found that Stat3 significantly increased the activity of the −438 to −474 bp *FANCC*/minimal promoter construct (*p* < 0.0001, *n* = 6) (Fig. 2*A*). Mutation of either consensus significantly decreased this activity (*p* < 0.001, *n* = 6), but activation by Stat3 was completely abolished by mutation of both (*p* = 0.6, *n* = 6 relative to minimal promoter/reporter control) (Fig. 2*A*). Stat5 had no effect on the −438 to −474 bp construct, and none of these proteins influenced the empty minimal promoter/luciferase reporter vector (Fig. 2*A*).

We identified a potential C/ebp binding site between −392 and −403 in the *FANCC* promoter (consensus, 5′-T[TG]NNGNNAA[TG]-3′; *FANCC* sequence, 5′-TAGGGGAAATC-3′) (26). We generated a minimal promoter/reporter construct with three copies of the −385 to −408 bp *FANCC* promoter sequence. An additional construct was generated with mutation of this consensus. Reporter activity was assayed in U937 cells co-transfected with vectors to express C/ebpβ-Lap, C/ebpβ-Lip, or control vector (and the internal control reporter vector described above). C/ebpβ-Lap increased the activity of the −385 to −408 bp *FANCC* sequence significantly (*p* < 0.0001, *n* = 6) (Fig. 2*B*). C/ebpβ-Lip did not activate this *FANCC* sequence, and neither protein activated the minimal promoter/reporter control vector.

We investigated binding of Stat3 and C/ebpβ to these *FANCC* promoter regions by chromatin co-immunoprecipitation. For these studies, lysates of Lin^−^CD34^+^ murine bone marrow cells were immunoprecipitated with antibodies to Stat3, C/ebpβ, or an irrelevant control antibody (27). Some cells were differentiated with IL1β for 24 h prior to cross-linking and lysis. Co-precipitating chromatin was amplified by semiquantitative PCR (Fig. 2*C*) or quantitative real-time PCR (using SYBR Green and the standard curve method) (Fig. 2*D*).

We found specific co-precipitation of the −380 to −410 bp sequence with C/ebpβ and of the −430 to −470 bp sequence by Stat3 (Fig. 2*C*). Co-precipitation of both proteins was significantly increased by IL1β. Neither protein co-precipitated the irrelevant 5′ flank sequence (data not shown).

### IL1β increased expression of FancC, Stat3, and C/ebpβ in murine bone marrow cells

Reporter assays in cell lines provide information regarding promoter activity but should be interpreted carefully because of the transformed nature of these cells. To investigate FancC expression in a non-transformed setting, and possible contributions by Stat3 or C/ebpβ, we performed studies in primary myeloid progenitor cells from murine bone marrow. For these studies, Lin^−^CD34^+^ cells were isolated, and some cells were differentiated with IL1β or G-CSF. The amount of G-CSF employed was consistent with serum levels in emergency granulopoiesis (9). Total cellular RNA was analyzed for gene expression by quantitative real-time PCR. The standard curve method was used, so data are presented as mRNA abundance according to this technique.

**Figure 3.**
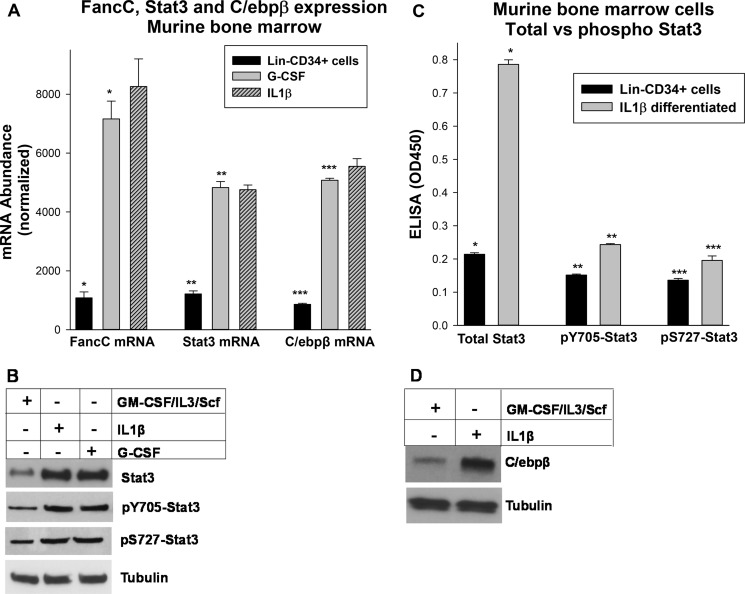
**Expression of Stat3, C/ebpβ, and FancC increased IL1β- or G-CSF–differentiated cells.** *A*, differentiation of murine bone marrow myeloid progenitor cells with IL1β or G-CSF increased Stat3, C/ebpβ, and FancC mRNA. Lin^−^CD34^+^ murine bone marrow cells were isolated, and some cells were differentiated with IL1β or G-CSF for 48 h prior to analysis. RNA expression was analyzed by real-time PCR. Statistically significant differences are indicated by *, **, or *** (*p* < 0.001, *n* = 6 for all comparisons). *B* and *C*, expression of total and phospho-Stat3 protein was increased by IL1β-differentiation. Lin^−^CD34^+^ cells were isolated from bone marrow mononuclear cells from the femora of mice. Some cells were differentiated with IL1β for 48 h prior to analysis. Cell lysates were analyzed by Western blot (*B*) or ELISA (*C*) for total Stat3, Tyr(P)-705–Stat3, or Ser(P)-727–Stat3. Statistically significant differences are indicated by *, **, or *** (*p* < 0.001, *n* = 6 for all comparisons). *D*, C/ebpβ-Lap protein was increased by IL1β differentiation of these cells. Cell lysates were also analyzed by Western blots serially probed with antibodies to C/ebpβ or tubulin (as a loading control). Representative blots are shown.

We found significantly increased FancC mRNA in response to treatment with either cytokine (*p* < 0.001, *n* = 4) (Fig. 3*A*). We also found a significant cytokine-induced increase in Stat3 and C/ebpβ mRNA in these cells (*p* < 0.001, *n* = 4) (Fig. 3*A*). The relative increase in expression of Stat3 and C/ebpβ in response to either IL1β or G-CSF was not significantly different (*p* = 0.2, *n* = 4).

We next investigated the impact on Stat3 protein. For these studies, cells were analyzed with or without IL1β or G-CSF treatment for total Stat3, Tyr(P)-705–Stat3, or Ser(P)-727–Stat3 by Western blot. We found that either cytokine increased total and phospho-Stat3 protein (Fig. 3*B*).

To quantify these results, we performed an ELISA for total Stat3, Tyr(P)-705–Stat3, or Ser(P)-727–Stat3. We found that IL1β also significantly increased total Stat3 protein in this assay (*p* < 0.001, *n* = 3) (Fig. 3*C*). Although phosphotyrosine or phosphoserine Stat3 increased, the relative increase was significantly less than in total Stat3 protein (4-fold increase *versus* ∼50% increase) (Fig. 3*C*).

We similarly investigated the impact of emergency granulopoiesis on C/ebpβ protein (by Western blot). We found that IL1β increased C/ebpβ expression, consistent with our mRNA studies (Fig. 3*D*). In this experiment, only the C/ebpβ-Lap form was detected (35 kDa), not the smaller Lip form (∼20 kDa).

### Abundance of Stat3 protein influenced FANCC promoter activity

We also investigated the roles of tyrosine or serine phosphorylation of Stat3 on activation of the *FANCC* cis element. For these experiments, we co-transfected U937 cells with a vector to express Y705F-Stat3 or S727A-Stat3 and the −482 to −517 bp *FANCC* minimal promoter/reporter vector (or control minimal promoter/reporter vector). Cells were also co-transfected with the internal control reporter vector as described above. Phosphorylation of Tyr-705 enhances transcriptional activation of some target genes by Stat3 (23). Phosphorylation of Ser-727 was found to either enhance or inhibit this effect in a context-dependent manner (25).

**Figure 4.**
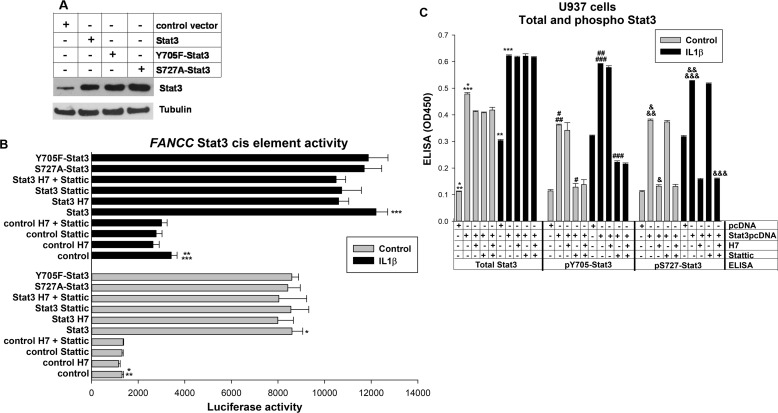
**Stat3 protein abundance contributes to *FANCC* promoter activation.** *A*, Stat3, Y705F-Stat3, and S727A-Stat3 are equivalently overexpressed in U937 cells. U937 cells were transfected with vectors to overexpress various Stat proteins. Protein expression was determined by Western blots serially probed with antibodies for Stat3, Tyr(P)-705–Stat3, Ser(P)-727–Stat3, or tubulin (as a loading control). A representative blot is shown. *B*, increased abundance of Stat3 protein increases the activity of the *FANCC* promoter Stat3-binding cis element. U937 cells were transfected with a minimal promoter/luciferase reporter construct with −438 to −474 bp of *FANCC* 5′ flank (or the minimal promoter/luciferase reporter control vector). Cells were co-transfected with vectors to overexpress Stat3, S727A-Stat3, or Y705F-Stat3 (*versus* control vector). Some transfectants were treated with a serine kinase inhibitor (H7), a tyrosine kinase inhibitor (Stattic), or both, and some were differentiated with IL1β prior to analysis. Statistically significant differences are indicated by *, **, or *** (*p* < 0.01, *n* = 6 for all comparisons). *C*, H7 or Stattic alter the Stat3 phosphorylation state but not protein abundance. U937 cells were transfected and assayed under the conditions described for reporter gene assays. Total, Tyr(P)-705–Stat3, or Ser(P)-727–Stat3 were analyzed by ELISA. Statistically significant differences are indicated by *, **, ***, #, ##, ###, &, &&, or &&& (*p* < 0.01, *n* = 6 for all comparisons).

In initial studies, we performed Western blots to verify overexpression of these forms of Stat3 in U937 cells. We found that WT, Y705F, or S727A Stat3 were equivalently expressed under the assay conditions (Fig. 4*A*).

We also found that this *FANCC* cis element was equivalently activated by tyrosine mutant, serine mutant, or WT Stat3 (*p* ≥ 0.2, *n* = 6) (Fig. 4*B*). We found that IL1β increased the activity of the *FANCC* cis element with or without Stat3 overexpression, consistent with the effect of increased endogenous Stat3 in IL1β-treated cells.

We further investigated the impact of Stat3 phosphorylation on *FANCC* cis element activity in assays using an inhibitor of Stat3 serine phosphorylation (H7), tyrosine phosphorylation (Stattic), or both. We found that neither inhibitor altered the effect of overexpressed Stat3 on the *FANCC* cis element, individually or in combination (*p* > 0.1, *n* = 6 for all comparisons) (Fig. 4*B*). We verified the effect of these inhibitors on Stat3 in control ELISA experiments (Fig. 4*C*). None of these forms of overexpressed Stat3, nor treatment with any of the inhibitors, influenced the activity of the control minimal promoter-reporter vector (data not shown).

### Phased expression of Stat3, C/ebpβ, and Icsbp sustains FancC expression during emergency granulopoiesis

We also investigated the impact of endogenous Stat3 or C/ebpβ on FancC expression in differentiating murine bone marrow cells. For these studies, Lin^−^CD34^+^ cells were transduced with retroviral vectors to express shRNAs specific to Stat3, C/ebpβ, or both. We tested scrambled shRNAs for each of these proteins as controls, and none influenced the target protein or FancC mRNA, so they were combined for these experiments. Some cells were treated with IL1β, and FancC mRNA was quantified, with quantification of Stat3 and C/ebpβ expression as controls.

**Figure 5.**
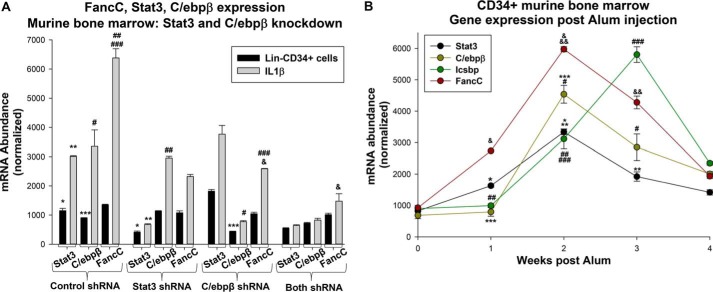
**Stat3 and C/ebpβ influence FancC expression during emergency granulopoiesis.** *A*, knockdown of Stat3 or C/ebpβ decreases IL1β-induced expression of Fanconi C. Murine bone marrow myeloid progenitor cells were transduced with vectors to express shRNAs specific for Stat3 or C/ebpβ (or scrambled control shRNA) and analyzed for FancC expression. Statistically significant differences are indicated by *, **, ***, #, ##, ###, or & (*p* < 0.01, *n* = 6 for all comparisons). *B*, expression of Stat3 and C/ebpβ increases early, Icsbp increases later, and FancC is increased throughout emergency granulopoiesis. Mice were injected with Alum (i.p.) to induce emergency granulopoiesis, and bone marrow Lin^−^CD34^+^ cells was collected 0, 1, 2, 3, or 4 weeks after injection. Gene expression was determined by quantitative real-time PCR. Statistically significant differences are indicated by *, **, ***, #, ##, ###, &, or && (*p* < 0.01, *n* = 6 for all comparisons).

We found that knockdown of either protein significantly impaired FancC expression in differentiating cells (*p* < 0.001, *n* = 3) (Fig. 5*A*). The combined effect of knocking down both Stat3 and C/ebpβ was greater than either alone (*p* < 0.001, *n* = 3) (Fig. 5*A*). Because the total amount of plasmid was kept constant, the combined amount of shRNA in samples with knockdown of both Stat3 and C/ebpβ was half as much as in experiments with either alone.

Stat3, C/ebpβ, and Icsbp may activate *FANCC* transcription at different times during emergency granulopoiesis. Specifically, Stat3 and C/ebpβ are involved in initiating and maintaining emergency granulopoiesis, whereas Icsbp terminates this process (in part by decreasing Stat3 and C/ebpβ expression) (13–15). We investigated this hypothesis using an *in vivo* murine model of emergency granulopoiesis.

For these studies, we induced emergency granulopoiesis in mice by i.p. injection of Alum or saline (as a control for steady-state granulopoiesis, *n* = 6 mice per group) (9, 15, 18). Alum injection results in maximal expansion of myeloid progenitor cells and differentiating granulocytes in the bone marrow by 2 weeks and resumption of the steady state by 4 weeks after injection (9, 15, 18). To investigate mRNA expression of FancC, Stat3, C/ebpβ, and Icsbp at various points during this process, cohorts of mice were sacrificed 0, 1, 2, 3, and 4 weeks after Alum injection, and Lin^−^CD34^+^ bone marrow cells were analyzed.

We found significantly increased expression of Stat3 and C/ebpβ that was maximal 2 weeks after Alum injection (*p* < 0.0001, *n* = 3 relative to the steady state) and began to decrease at 3 weeks (*p* < 0.001, *n* = 3 for comparison of 1 *versus* 2 or 2 *versus* 3 weeks) (Fig. 5*B*). In contrast, Icsbp mRNA was maximally expressed 3 weeks after Alum injection (*p* < 0.001, *n* = 3 for comparison of 2 and 3 weeks) and was returning to steady-state levels at 4 weeks (Fig. 5*B*). FancC mRNA expression was significantly increased 1 week after Alum injection, maximal at 2 weeks (*p* < 0.0001, *n* = 3), and returning to baseline at 4 weeks (Fig. 5*B*).

### Stat3 or C/ebpβ rescues DNA repair in a FancC-dependent manner

We were interested in determining the impact of Stat3 or C/ebpβ on DNA repair in cells exposed to the stress of emergency granulopoiesis. To investigate this, we used a plasmid-based DNA repair assay we employed previously to study the influence of Icsbp on this process. In this study, a reporter plasmid is treated with mitomycin C to generate DNA cross-links (with an untreated plasmid as a control) (9, 17). Damaged or undamaged reporter plasmids are transfected into U937 cells, and reporter activity represents the efficiency of DNA repair. Cells are co-transfected with a second reporter plasmid as an internal control for transduction efficiency (not MMC-treated).

In prior studies, we determined that U937 cells efficiently repaired the MMC-treated plasmid, but treatment of U937 cells with differentiating agents (retinoic acid/dimethyl formamide or Ifnγ) significantly impaired this activity (9, 17). For this study, U937 cells were co-transfected with an MMC-treated or untreated control plasmid (CMV-firefly luciferase vector) and vectors to overexpress Icsbp, Stat3, C/ebpβ-Lap, combinations of these proteins, or empty control expression vector (with the TK-*Renilla* luciferase vector as a control for transfection efficiency). Transfectants were assayed after 24 h of treatment with IL1β.

**Figure 6.**
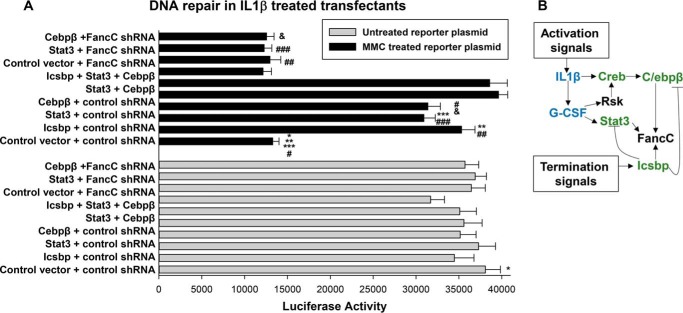
**Stat3 and C/ebpβ enhance DNA repair in a FancC-dependent manner in myeloid cells undergoing IL1β-induced differentiation.** *A*, Icsbp, Stat3, and C/ebpβ rescue DNA repair during IL1β-induced differentiation of U937 cells, but this is reversed by FancC knockdown. U937 cells were co-transfected with a mitomycin C (MMC) cross-linked luciferase reporter plasmid (or untreated control luciferase reporter plasmid) or vectors to express Icsbp, Stat3, or C/ebpβ alone or in combination (or control vector). Other cells were transfected with vectors to express Icsbp, Stat3, or C/ebpβ and express FancC-specific shRNAs (or scrambled control shRNA). Some transfectants were treated for 24 h with IL1β before reporter activity was determined. Statistically significant differences are indicated by *, **, ***, #, or ## (*p* < 0.001, *n* = 6 for all comparisons). *B*, schematic of the regulation of FancC expression by Icsbp, Stat3, and C/ebpβ during emergency granulopoiesis. Cross-regulation of these transcription factors is also indicated.

We found significantly less firefly luciferase reporter activity from the MMC-treated reporter vector compared with the untreated control reporter vector in IL1β-treated transfectants (*p* < 0.001, *n* = 6) (Fig. 6*A*). Overexpression of Icsbp, Stat3, or C/ebpβ significantly increased the reporter activity of the MMC-treated plasmid in IL1β transfectants (*p* < 0.001, *n* = 6) but had no effect on the activity of reporter vectors that had not been MMC-treated (Fig. 6*A*). Assays with combinations of the three proteins demonstrated that effects were non-redundant because the total amount of expression plasmid was held constant in these experiments. In the absence of IL1β treatment of the transfectants, we found that the luciferase reporter activity from the MMC-treated plasmid was not significantly different than the activity of the untreated plasmid (data not shown), consistent with our prior studies (9, 17).

We were interested in determining whether the effects of Icsbp, Stat3, or C/ebpβ on the activity of the MMC-damaged reporter plasmid required FancC expression. To examine this, we co-transfected U937 cells with MMC-treated or an untreated reporter plasmid; vectors to overexpress Icsbp, Stat3, or C/ebpβ; vectors to express shRNAs specific to FancC (or scrambled shRNA control); and a plasmid to control for transfection activity (as above). Transfectants were assayed after 24-h treatment with IL1β.

We found that knockdown of FancC prevented Icsbp, Stat3, or C/ebpβ overexpression from rescuing the reporter activity of the MMC-treated plasmid (Fig. 4*A*). These results suggested that these transcription factors were acting through a FancC-dependent mechanism to drive DNA cross-link repair.

## Discussion

Emergency granulopoiesis is a high-risk/high-gain response to infectious challenge. During this process, rapid granulocyte production requires increased proliferation and faster differentiation of bone marrow stem and progenitor cells, resulting in genotoxic stress. This risk is further enhanced by the apoptosis resistance and cell cycle shortening that occur during this process (9, 15). In this study, we found Stat3 and C/ebpβ, essential transcription factors for initiating and sustaining emergency granulopoiesis, are involved in protecting the genome by increasing the expression of Fanconi C.

We also found that Stat3, C/ebpβ, and Icsbp are non-redundant for activation of the *FANCC* promoter and that IL1β increases *FANCC* promoter activity and enhances the effects of these transcription factors. Also, our study demonstrates that IL1β increases binding of Stat3 and C/ebpβ to their respective *FANCC* cis elements, perhaps because of the ability of IL1β to increase the expression of Stat3 and C/ebpβ mRNA and protein. We found that IL1β treatment decreases the ability of U937 myeloid cells to repair DNA cross-links and that DNA cross-link repair is rescued by Stat3 or C/ebpβ in a FancC-dependent manner, similar to our prior results with Icsbp and FancC (9).

Our studies suggest that different expression levels of Stat3, C/ebpβ, and Icsbp at various times during emergency granulopoiesis ensure sustained FancC expression throughout this process. At initiation of emergency granulopoiesis, Stat3 expression increases, followed by expression of C/ebpβ, correlating with increased FancC expression. All three transcription factors are expressed at peak granulocyte production during Alum-stimulated emergency granulopoiesis, correlating with maximal FancC expression and consistent with maximal protection from DNA damage at this point in the process.

We found that expression of FancC was still increased, relative to the steady state, 3 weeks after initiation of emergency granulopoiesis, despite decreasing Stat3 and C/ebpβ at this time. However, Icsbp expression was persistently elevated at this point. In prior studies, we found that emergency granulopoiesis failed to terminate in Icsbp^−/−^ mice, associated with increased and sustained Stat3 and C/ebpβ expression (15). This implicated Icsbp in resetting Stat3 and C/ebpβ to steady-state levels. These results determined that Icsbp antagonizes FancC expression by decreasing Stat3 and C/ebpβ but compensates for this effect by activating the *FANCC* promoter until resumption of the steady state (Fig. 5*B*) (15, 22).

We found that Stat3, C/ebpβ, and Icsbp each activate different *FANCC* promoter cis elements. We previously found that activation of the *FANCC* promoter by Icsbp was enhanced by tyrosine phosphorylation of this protein during emergency granulopoiesis, identifying roles for enhanced expression and posttranslational modification in this process (9). In this study, we found that neither tyrosine nor serine phosphorylation of Stat3 enhances *FANCC* promoter activation, suggesting that protein expression is driving function. This is in contrast to some other Stat3 target genes, where tyrosine phosphorylation enhances transcriptional activation (23). The role of Ser(P)-727 in Stat3 function is controversial and may be context-dependent (25).

In addition to regulating *FANCC* transcription, Icsbp also enhances calpain activity through repression of the growth-specific arrest 2 (*GAS2*) gene, a calpain inhibitor (28). Because Stat3 is a calpain substrate, Icsbp may influence both Stat3 mRNA expression and Stat3 protein stability during termination of emergency granulopoiesis (29). Understanding cooperation *versus* antagonism between Icsbp and Stat3 is of interest to understand the innate immune response and a focus of ongoing investigations in the laboratory.

The Stat3-binding *FANCC* cis element identified in this work has a tandem binding consensus sequence, and we found that both copies were required for maximal cis element activity. This is consistent with interaction of Stat3 as a homodimer with such tandem binding sites in a number of pro-inflammatory genes (30).

Although this cis element was activated by Stat3, we found no effect of Stat5 on the *FANCC* promoter. Conversely, we found previously that Stat5 represses the distal *IRF8* promoter in myeloid progenitor cells (31), but Stat3 had no effect. We also determined previously that Icsbp regulates Stat5 protein stability (through Gas2/calpain) but does not influence Stat5 mRNA (31). Therefore, these two Stat proteins play discrete roles during myelopoiesis and are differentially regulated by Icsbp.

We found that the C/ebpβ Lap isoform activated a cis element in the *FANCC* promoter but that the Lip isoform did not. These isoforms were originally described in regenerating liver cells, and Lip (liver inhibitory protein) antagonized the effects of Lap (liver activating protein) in these cells (19). In our study, we found that Lap was the dominant C/ebpβ isoform in myeloid cell lines and differentiating murine bone marrow progenitors. Overexpression of Lip did not repress *FANCC* promoter activity in myeloid cell line transfectants, but we also did not find an increase in Lip during termination of emergency granulopoiesis. This suggests that other mechanisms, such as general repression of *CEBPB* transcription by Icsbp-dependent events, may regulate C/ebpβ activity during emergency granulopoiesis.

Increased expression of Stat3 and C/ebpβ is found in chronic myeloid leukemia (CML) (32, 33). The function of these transcription factors in leukemogenesis may be consistent with their normal roles in expanding myeloid progenitor populations during emergency granulopoiesis. In contrast, Icsbp is a leukemia suppressor for CML, with decreased expression in this disease (34, 35). Therefore, regulation of emergency granulopoiesis may represent a paradigm for leukemia promotion *versus* suppression.

It is additionally possible that episodes of emergency granulopoiesis facilitate leukemogenesis under conditions with decreased Icsbp or enhanced expression of Stat3 or C/ebpβ. All three proteins enhance FancC expression, which would be anticipated to protect cells from DNA damage. However, we found significantly more *FANCC* promoter activity and FancC expression in the presence of all three transcription factors compared with Stat3 and C/ebpβ without Icsbp. It would be of interest to determine whether repeated episodes of emergency granulopoiesis enhance drug resistance or progression to blast crisis in CML. Studies are currently being performed in the laboratory to address this issue.

## Experimental procedures

### Protein expression vectors

The Icsbp/Irf8 cDNA was obtained from Dr. Ben Zion-Levi (Technion, Haifa, Israel) and subcloned into the mammalian expression vector pcDNA (Stratagene, La Jolla, CA), as described previously (36). Wildtype and Y705F mutant murine Stat3 cDNAs and C/ebpβ Lap and Lip cDNAs were obtained from Addgene and subcloned into the pcDNA (for expression in myeloid cell lines) and MSCV (for generation of retrovirus) vectors. FancC-specific shRNAs (and scrambled control shRNAs) were generated using the Promega website and subcloned into the pLKO retroviral vector.

### Reporter constructs

The human *FANCC* 5′ flank (1.0 kb from the ATG codon) was generated by PCR from the U937 myeloid cell line. The genomic clone was sequenced to ensure identity with the sequence in the ENSEMBL database (37). This sequence and additional truncations (−500, −450, or −400 bp) were subcloned into the pGL3-basic reporter vector (Promega, expressing the firefly luciferase reporter gene). Other constructs were generated with one copy of the −387 to −403 bp *FANCC* promoter or with three copies of the −470 to −530 bp *FANCC* promoter subcloned into a minimal promoter-reporter vector (pGL3-promoter vector) (Promega, with the TK minimal promoter expressing firefly luciferase reporter gene). Some of the −387 to −403 bp *FANCC* promoter/minimal promoter/reporter constructs had mutation of the proximal, distal, or both Stat consensus binding sequences.

### Myeloid cell line culture

The human myelomonocytic leukemia cell line U937 (38) was obtained from Andrew Kraft (University of Arizona, Tucson, AZ). Cells were maintained as described previously (38).

### Transfections and reporter gene assays

U937 cells were transfected with *FANCC* promoter/luciferase reporter constructs (or empty control reporter vector) and various combinations of vectors to overexpress Stat3, C/ebpβ, or Icsbp (or empty expression vector) or specific shRNAs to knock down Stat3 or C/ebpβ (or scrambled shRNA control vectors). Cells were also co-transfected with an internal control plasmid to normalize for transfection efficiency (Promega Dual-Luciferase system, a CMV-luciferase reporter vector expressing *Renilla* luciferase). Transfectants were assayed for luciferase activity according to the instructions of the manufacturer. Some transfectants were treated with IL1β (50 ng/ml for 24 h) prior to harvesting.

Luciferase activity from control empty reporter vectors was not influenced by IL1β treatment or overexpression or knockdown of any of these proteins and was subtracted as background. All reporter assays were repeated six times in independent experiments (and samples were assayed in duplicate) for each condition.

The efficacy of IL1β differentiation of U937 cells was verified for various batches of cytokines used in these studies by determining enhanced *FANCC* promoter activity in transfection assays or expression of endogenous mRNA for FancC and gp91phox in cells treated with the cytokine. Some transfectants were studied after treatment for 24 h with H7 (a serine kinase inhibitor for Stat3 (40)) or Stattic (a specific inhibitor of Stat3 tyrosine phosphorylation (41)).

### Mitomycin C treatment of plasmids and DNA repair assays

To generate DNA cross-links, purified plasmid DNA (CMV-firefly luciferase from Promega) was incubated with mitomycin C (40 μm) for 12 h at room temperature. Plasmid DNA was recovered by phenol:chloroform extraction followed by ethanol precipitation. DNA cross-linking was verified by non-denaturing agarose gel electrophoresis (17).

U937 cells were co-transfected with cross-linked or untreated control reporter plasmid, vectors to overexpress Icsbp, Stat3, or C/ebpβ (or control expression plasmid), and vectors expressing specific shRNAs to knock down FancC (or scrambled control). Cells were also transfected with the TK-*Renilla* luciferase vector as an internal control for transfection efficiency. Lysates were analyzed and simultaneously assayed for dual luciferase activity as described above. Luciferase reporter activities were determined after 24-h treatment with IL1β. Reporter assays were repeated six times in duplicate as described above.

### Western blot and ELISA of lysate proteins

For Western blots, cells were lysed by boiling in 2× SDS sample buffer. Lysate proteins (50 μg) were separated by SDS-PAGE (10% acrylamide) and transferred to nitrocellulose, and filters were serially probed with antibodies as described previously (39). Each experiment was repeated at least three times with different sets of lysates, and a representative blot is shown.

In other experiments, total, serine-phosphorylated, or tyrosine-phosphorylated Stat3 proteins in cell lysates were quantified by commercially available ELISA (Abcam, Cambridge, MA). ELISAs were performed in duplicate on three independent sets of lysates, and the results were graphed as *A*_450_ (according to the instructions of the manufacturer).

### Chromatin immunoprecipitation

Cells were incubated briefly in medium supplemented with formaldehyde, and lysates were sonicated to generate chromatin fragments with an average size of 500 bp and immunoprecipitated with Stat3, C/ebpβ, or irrelevant control antibody (Abcam) (26). Chromatin was amplified by quantitative real-time PCR using SYBR Green and the standard curve method (Thermo Fisher Scientific) according to the instructions of the manufacturer). Primers were designed flanking the Stat3 or C/ebp consensus sequences in the *FANCC* promoter. The standard curve was generated using total chromatin from murine bone marrow cells. Input chromatin (not precipitated) from each sample was analyzed to normalize data between the samples. At least three independent immunoprecipitation experiments were performed, and the samples were analyzed in triplicate.

### Quantitative real-time PCR

RNA was isolated using TRIzol reagent (Gibco-BRL, Gaithersburg, MD) and tested for integrity by denaturing gel electrophoresis. Primers were designed with Applied Biosystems software, and real-time PCR was performed using SYBR Green and the standard curve method. The standard curve for these experiments was generated with cDNA from WT cells cultured in GM-CSF, IL3, and stem cell factor. The results were normalized to 18S and actin and presented as mRNA abundance with 1 ng of cDNA set as 1000 in the standard curve. At least three independent samples were evaluated in triplicate.

### Animal use

The mice used for this study were C57 Black 6 and maintained in an approved and accredited animal facility at Northwestern University. The mice were housed in a specific pathogen-free, tightly regulated environment that included control of the flow of animals, equipment, and personnel and use of micro-isolator cages and husbandry procedures to minimize pathogen exposure and disease outbreak. All work was reviewed and approved by the Animal Care and Use Committees of Jesse Brown Veterans Affairs Medical Center and Northwestern University.

### In vitro murine studies

For *in vitro* studies, bone marrow mononuclear cells were harvested by flushing femora repeatedly with Hanks' balanced salt solution until no additional cells were obtained. Washed cells were treated with ammonium–chloride–potassium buffer to lyse red blood cells and then washed extensively. Lin^−^CD34^+^ cells were separated using a magnetic bead–based, affinity chromatography–based technique according to the instructions of the manufacturer (Miltenyi Biotech, San Diego, CA). Cells were cultured (2 × 10^5^/ml) for 48 h in Dulbecco's modified Eagle's medium supplemented with 10% fetal calf serum, 1% penicillin-streptomycin, 10 ng/ml murine GM-CSF (R&D Systems Inc., Minneapolis, MN), 10 ng/ml murine recombinant IL3 (R&D Systems Inc.), and 100 ng/ml of stem cell factor (R&D Systems Inc.). Cells were maintained in GM-CSF, IL3, and stem cell factor for 24 h or stimulated with 50 ng/ml G-CSF (R&D Systems Inc.) or 20 ng/ml IL1β (R&D Systems Inc.) during this time period. Apoptotic cells were removed before analysis according to the instructions of the manufacturer (Miltenyi, Dead Cell Clean Up). Some cells were transduced with retroviral vectors prior to analysis according to techniques described in our prior work (9).

### In vivo murine emergency granulopoiesis assay

WT mice (18–20 weeks of age) were injected i.p. with Alum or saline control (12 mice per group). Mice were randomly assigned to cohorts for injection of Alum or saline control. Alum was prepared as described previously (9, 15, 18), and a volume of 0.5 ml was injected.

Cohorts of mice (6 per group) were sacrificed weekly, and bone marrow was collected from both femora. Successful induction of emergency granulopoiesis in Alum-injected mice (compared with saline control) was verified by weekly peripheral blood granulocytes counts (using a Hemavet automated cell counter; Drew Scientific, Miami Lakes, FL). Blood count data were analyzed by an investigator who was blinded to the status of the mice as Alum *versus* saline injected.

### Statistical analysis

Statistical significance was determined by unpaired two-tailed Student's *t* test (comparing two conditions) or analysis of variance (for more than two conditions) using SigmaPlot software. *p* < 0.02 was considered statistically significant. In all graphs, *error bars* represent ± S.E.

## Author contributions

C. A. S., L. Broglie, L. H., L. Bei, W. H., and D. B. D. investigation; C. A. S., L. H., W. H., and E. A. E. methodology; W. H. and E. A. E. conceptualization; E. A. E. formal analysis; E. A. E. supervision; E. A. E. funding acquisition; E. A. E. project administration.

## Footnotes

3 The abbreviations used are:

FA: Fanconi anemia
BMF: bone marrow failure
IL: interleukin
GM-CSF: granulocyte/macrophage colony-stimulating factor
i.p.: intraperitoneal(ly)
CMV: cytomegalovirus
shRNA: short hairpin RNA
MMC: mitomycin C
CML: chronic myeloid leukemia
TK: thymidine kinase
G-CSF: granulocyte colony stimulating factor.
